# The L1 spino-pelvic (L1SP) angle: a simplified approach for the assessment of the PI-LL mismatch in hip surgery

**DOI:** 10.1177/11207000241282984

**Published:** 2024-09-23

**Authors:** A Mounir Boudali, Yuan Chai, John E Farey, Jonathan Vigdorchik, William L Walter

**Affiliations:** 1 Sydney Musculoskeletal Health and The Kolling Institute, Northern Clinical School, Faculty of Medicine and Health and the Northern Sydney Local Health District, Sydney, NSW, Australia; 2 Institute of Future Health, South China University of Technology, Guangzhou, China; 3 Department of Orthopaedics and Traumatic Surgery, Royal North Shore Hospital, St Leonards, NSW, Australia; 4 Adult Reconstruction and Joint Replacement Service, Hospital for Special Surgery, New York, NY, USA

**Keywords:** Hip arthroplasty, lumbar lordosis, pelvic incidence, PI-LL mismatch, spinopelvic alignment

## Abstract

**Introduction:**

Pelvic incidence - lumbar lordosis (PI-LL) mismatch is often considered when assessing spinopelvic alignment in the sagittal plane. The mismatch is conventionally obtained by measuring 2 separate angles on lateral spinopelvic radiographs. This study describes a simplified approach for assessing spinopelvic mobility and measuring the PI-LL mismatch through the evaluation of the L1-spinopelvis angle (L1SP).

**Methods:**

96 standing lateral radiographs were obtained from consecutive patients presenting for total hip arthroplasty between November 2020 and July 2021. 3 operators were recruited to annotate landmarks on digital radiographs. Correlation analysis and error analysis were applied. Measurement reproducibility was assessed using intraclass correlation coefficient (ICC).

**Results:**

The correlation coefficients of the 3 variables were respectively 0.87 for PI, 0.94 for LL, and 0.96 for L1SP. The normalised root mean square error between the 2 measurement sets was 9.96% for PI, 5.97% for LL, and 4.41% for L1SP. The absolute error was 3.49° ± 4.63° for PI, 3.23° ± 3.78° for LL, 2.68° ± 3.19° for PI-LL conventional, and 2.35° ± 2.88° for PI-LL via L1SP, respectively. In terms of reproducibility, measurement of L1SP outperformed that of PI and LL (ICC = 0.97 versus 0.83 and 0.93, respectively).

**Conclusion:**

The simplified L1SP method, through the measurement of a single angle, produced similar measurements to the conventional PI-LL method. The measurement repeatability between operators was improved using the L1SP method. From a clinical practice perspective, both methods are equivalent. The new method is readily reproducible using commercially available PACS software during preoperative templating.

## Introduction

Spino-pelvic alignment represents the intricate relationship between the spine and the pelvis. It plays a pivotal role in both spine and hip surgeries.^1–5^ The alignment of these 2 anatomical regions has been increasingly recognised as a critical factor influencing surgical outcomes and patient satisfaction.^6,7^ Proper spino-pelvic alignment is important in maintaining an energy-efficient posture and ensures even distribution of forces and overall spinal balance leading to improved patient outcomes.^8,9^ In spine surgery, maintaining appropriate spino-pelvic alignment is crucial for achieving successful spinal fusion, mitigating adjacent segment degeneration, and improving patient postoperative function.^8^

Furthermore, the spino-pelvic alignment plays a crucial role in hip arthroplasty as it directly affects the stability and longevity of the prosthetic joint. An abnormal spino-pelvic mobility is associated with both dislocation and revision for dislocation in spinal spondylosis (stiff spine) prior to lumbar fusion surgery, especially at the L5-S1 level.^10,11^ Patients with such condition experience restrictions in their spine mobility, including limitations in bending and twisting. These restrictions can increase recruitment of the hip, causing additional stress on the soft tissue, which may contribute to THA instability, to premature wear of implants, and/or to an increased risk of hip dislocation. Therefore, surgeons should determine whether adjustments to acetabular cup positioning or component selection (e.g. dual-mobility implant design) should be made to compensate for spinal stiffness to mitigate the risks aforementioned.

Spino-pelvic alignment can be measured in terms of the mathematical difference between pelvic incidence (PI) and lumbar lordosis (LL) often referred to as PI-LL mismatch.^12,13^ This angle is important in spinal^12,14–17^ and hip^10,18–20^ arthroplasty. During preoperative templating for THA, surgeons may incorporate the PI-LL mismatch in their assessment of the global sagittal alignment.

Currently, evaluation of this angle requires the measurement of 2 independent angles (PI and LL), 1 of which is often visually located outside the standing lateral spine-pelvis-hip radiograph film. This makes it cumbersome when surgeons use physical radiographs during preoperative templating. Furthermore, the accumulation of operator error from the manual measurement of 2 separate angles could impact the outcome of the surgeon’s decision. With the increasing complexity and time demand of preoperative templating, simplification is needed without jeopardising the quality, accuracy, and reliability of the surgeon’s decisions.

We introduce the L1-spino-pelvic angle (L1SP), a simplified method of assessing the spino-pelvic mobility and evaluating the PI-LL mismatch from lateral x-rays. This angle aims to provide a comparable and potentially more reproducible alterative to the conventional PI-LL mismatch angle.

## Materials and methods

This retrospective study recruited 126 radiographs obtained using EOS stereographic system (EOS Imaging,Paris, France) from 99 consecutive patients who visited our institute between November 2020 and July 2021.^21^ Patients involved in this study provided written consent for using their de-identified data for research purposes. The storage, transfer, and use of data were ethically approved by the St. Vincent’s Hospital Human Ethics Committee (Ethics No. 2019/ETH09656). De-identified imaging data and parameter data are deposited at Figshare for research non-identifiable purposes only (10.6084/m9.figshare.23938398).

Radiographs in which the distance between the centre of the femoral heads was greater than the smallest femoral head diameter were deemed highly rotated pelvises and hence excluded from this study. Radiographs not capturing L1 were also excluded. 3 operators were recruited to perform landmark selection, 1 of whom performed the selection twice, with a 2-week period between landmark selection sessions.

**Figure 1.**
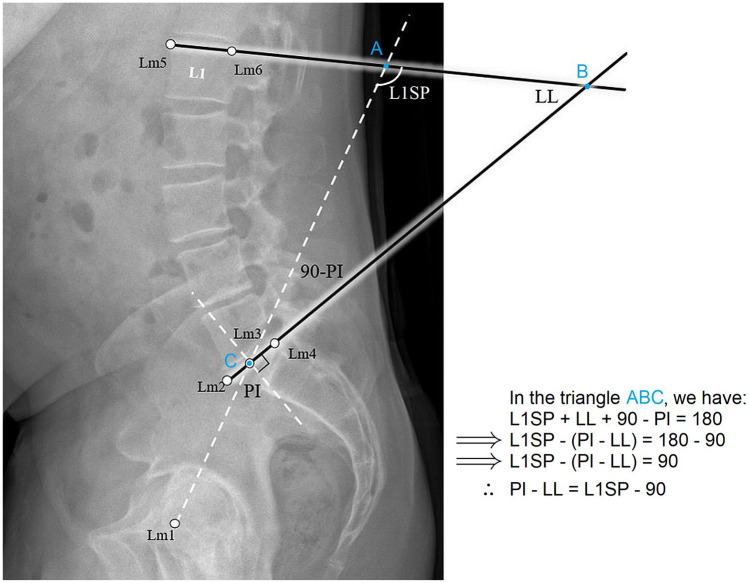
Illustration showing the simplified approach of measuring the PI-LL mismatch, and the landmarks (Lm 1–6) used to measure the pelvic incidence PI, the lumbar-lordosis LL, and L1 spinopelvic angle L1SP.

The landmarks selection consists of defining 6 landmarks (Figure 1) on the radiograph images as follows:

- 1 landmark at the midpoint of the segment defined by the center of the femoral heads
- 2 landmarks at the anterior and posterior margins of the superior S1 endplate
- 1 landmark at the midpoint of the superior S1 endplate
- 2 landmarks at the anterior and posterior margins of the superior L1 endplate

The operators performed this task using a custom script developed in the MATLAB platform (MATLAB R2021a. The MathWorks Inc., Natick, MA, USA). The landmarks’ coordinates were recorded and used to extract the angles PI, LL, and L1SP, as shown in Figure 1. Here, L1SP is the supplementary of the angle defined by 2 lines; a line passing through the centre of the femoral head axis and the centre of the S1 endplate, and a line tangential to the superior L1 endplate, as demonstrated in Figure 1.

We used the conventional method of estimating the PI-LL mismatch, computed as follows:

PI−LLmismatch=PI−LL

Extending the line defining the PI angle until the L1 endplate and drawing the lines defining LL angle creates a triangle (Figure 1). The sum of the interior angles of this triangle is 180°. Hence L1SP + LL + 90° - PI = 180°. After rearranging the variables in this equation, we have found:

PI−LLmismatch=L1SP−90°

To assess the reliability of the method, we used Pearson correlation coefficients between the different operators’ measurement sets. The correlation was considered negligible 0.00–0.10, weak 0.10–0.39, moderate 0.40–0.69, strong 0.70–0.89 and very strong 0.90–1.00.^22^ Repeatability was assessed using normalised root mean square error (NRMSE) values, defined as the root mean square of the error between 2 operators divided by the maximum range of their measurements for a given variable. NRMSE is more adequate in comparing datasets representing variables with different scales, and lower NRMSE values indicate less residual variance.

A 2-way mixed intraclass correlation coefficient (ICC) at 95% confidence interval (CI) was used to qualify the inter-operator reliability. ICC was considered as follows: <0.5 poor, 0.5–0.75 moderate, 0.75–0.9 good and 0.9–1.0 excellent.^23^

## Results

**Figure 2.**
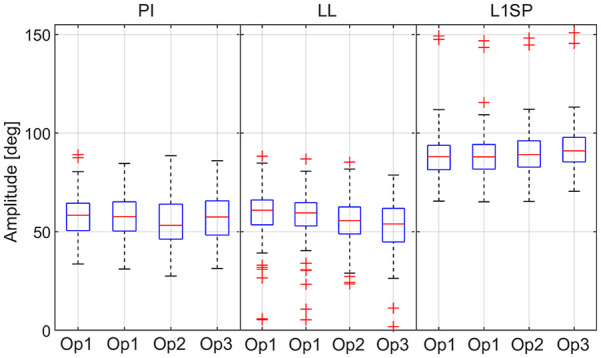
Boxplot of the measurements. The red line (across box) indicates the median, and the bottom and top edges of the box indicate the 25^th^ and 75^th^ percentiles, respectively. The whiskers extend to the range of data points not considered outliers. The latter are plotted using the ‘+’ symbol.

Amongst the images retrieved, 5 were excluded due to highly rotated pelvises, 24 due to superior L1 endplate outside the radiograph, and 1 duplicate. Hence, 96 radiographs representing 48 males and 28 females were analysed in this study. Note that a few patients have >1 radiograph corresponding to pre- and post-surgery. The recruited radiographs underwent measurement of PI (range 27.5° to 89.1°), LL (range 1.9–88.4°), and L1SP (range 65.3–150.8°) by 3 operators. The boxplot in Figure 2 shows the measurements recorded by all the operators. Compared to the simplified method (mean 0.0° ± 13.1), no difference was observed between the PI-LL mismatch from the conventional method (mean 0.22° ± 13.6; *p* > 0.05).

**Figure 3.**
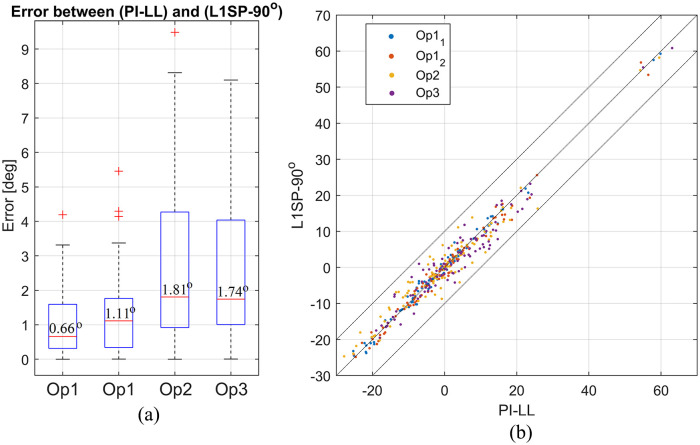
(a) Boxplot of the error between both methods for all operators. (b) Scatter plot showing PI-LL from the conventional method plotted against L1SP-90°.

The median absolute error between both methods was 0.9° for operator 1, 1.8° for operator 2, and 1.7° for operator 3 (Figure 3(a)). The overall absolute error between both methods has a mean of 1.9° ± 1.8° reflecting an excellent agreement between the methods. The latter is confirmed using a scatter plot, as presented in Figure 3(b). The overall average NRMSE error for PI-LL mismatch across all 3 operators was 6.5% (mean −2.9° ± 4.9°). This value increases to 7.53% (mean −3.3° ± 6.0°) for PI-LL measure using the conventional method, confirming that the simplified method of measuring PI-LL reliably produced similar measurements to the conventional method.

We examined intra-operator repeatability using data from 2 sets of measurements done by a single operator. The second set of landmarks selection happened 2 weeks after the first measurements. We found strong to very strong correlations between the 2 sets. The correlation coefficients of the 3 variables were respectively 0.87 for PI, 0.94 for LL, and 0.96 for L1SP. Furthermore, the NRMSE between the 2 sets of measurements was 9.96% for PI, 5.97% for LL, and 4.41% for L1SP. The absolute error between the 2 sets of measurements shows the same trend. For instance, the absolute error was 3.49° ± 4.63° for PI, 3.23° ± 3.78° for LL, 2.68° ± 3.19° for PI-LL conventional, and 2.35° ± 2.88° for PI-LL via L1SP, respectively. This indicates that the measurement of L1SP is more repeatable than PI and LL separately.

The inter-item ICC of the conventional method demonstrated a *good* to excellent inter-operator reliability (0.84–0.94), and the simplified method exhibited excellent repeatability among all comparisons (0.91–0.96), as summarised in Table 1. The ICC from the average measures showed that both methods had excellent reproducibility (PI-LL using the conventional method ICC = 0.95, 95% CI, 0.92–0.97, and L1SP-90° using the simplified method ICC = 0.97, 95% CI, 0.94–0.98). The ICC was 0.84, 0.93, 0.95, and 0.97 for PI, LL, PI-LL, and L1SP, respectively. The inter-operator reproducibility of LL and L1SP are excellent, and PI is good.

**Table 1.** 2-way mixed Inter-Item Correlation Matrix at 95% confidence interval applied to 3 operators - Op1, Op2, and Op3.

	*Conventional method*			*Simplified method*		
	Op1	Op2	Op3	Op1	Op2	Op3
*Op1*	-	0.943	0.889	-	0.962	0.926
*Op2*	0.943	-	0.839	0.962	-	0.913
*Op3*	0.889	0.839	-	0.926	0.913	-

## Discussion

A study reported that 58% of hip dislocations following a THA occur while the acetabular cup is positioned in the static Lewinnek Safe Zone.^24^ This confirms that the “one-size-fits-all” approach is ineffective and outdated. It is consequently important to consider the spine-pelvis-hip complex relations when planning for a functional cup positioning.

Following the PI-LL classification, the spine is said to be normal when PI-LL < 10°, the sagittal spinal deformity – or flatback deformity – is mild when 10° < PI-LL < 20°, and severe when PI-LL > 20° (Table 2).^25^ By analogy, the same classification will apply when considering the L1SP angle as a metric: normal when L1SP < 100°, mild when 100° < L1SP < 110°, and severe when L1SP > 110°, as summarised in Table 2. Patients with severe flatback deformity have higher risks of developing post-surgery complications such as instability and dislocation. They tend to recruit their pelvis more than their spine in situations requiring hip hyperflexion or internal rotation of the flexed hip (e.g., sit to stand manoeuver from a low position). It is therefore critical to preoperatively identify such patients to plan the optimal cup orientation and selection of implant and surgical approach.

**Table 2.** Flatback deformity classification based on the PI-LL mismatch and the L1SP angle.

Flatback Deformity	PI-LL	L1SP
Normal	<10°	<100°
Mild	10°< <20°	100°< <110°
Severe	>20°	>110°

Our results show that the simplified L1SP method produced similar measurements of PI-LL mismatch to the conventional method. Surgeons adopting our method should expect an improvement in the reliability and repeatability of the measurements, and an enhanced ability to assess the mismatch through the visualisation of the angle L1SP.

This study may have a few limitations. Due to time constraints and workability reasons, we limited the number of unique operators to 3. However, measurements done by additional operators are expected to show comparable results. We understand that using a MATLAB script to perform the measurements could be considered a limitation. Compared to available PACS software, the main difference resides in the landmark selection process. In MATLAB, operators are tasked to pinpoint the landmarks, whereas in a PACS software, operators can click and drag to draw lines, which can then be moved to pass by the relevant landmarks. An assessment using an available PACS software is required to validate the proposed method.

Often, surgeons do not have access to elaborate software that can allow advanced measurements on radiographs.^7^ The fact that the angle L1SP is always inside the limits of the radiograph makes the simplified method convenient for evaluating PI-LL manually on radiographic films. Other than hip surgeons, this method could also benefit spine surgeons in their spino-pelvic mobility assessment.

In conclusion, the proposed method consolidates the assessment of the spino-pelvic alignment and provides a simplified and particle way of measuring the PI-LL mismatch. The results suggest that the measured L1SP angle was the most repeatable amongst the 3 measured variables (PI, LL, L1SP). The simplified method offers a classification comparable to the conventional method for assessing the PI-LL mismatch, and it can be easily replicated using PACS software in hip arthroplasty pre-operative templating.
